# The Chemical Form of Metal Species Released from Corroded Taper Junctions of Hip Implants: Synchrotron Analysis of Patient Tissue

**DOI:** 10.1038/s41598-017-11225-w

**Published:** 2017-09-08

**Authors:** Anna Di Laura, Paul D. Quinn, Vasiliki C. Panagiotopoulou, Harry S. Hothi, Johann Henckel, Jonathan J. Powell, Fitim Berisha, Fernanda Amary, J. Fred W. Mosselmans, John A. Skinner, Alister J. Hart

**Affiliations:** 1 Institute of Orthopaedics and Musculoskeletal Science, University College London and the Royal National Orthopaedic Hospital, Stanmore, United Kingdom; 2 Diamond Light Source, Harwell Science and Innovation Campus, Didcot, United Kingdom; 3 Biomineral Research Group, Dept Veterinary Medicine, University of Cambridge, Cambridge, United Kingdom

## Abstract

The mechanisms of metal release from the articulation at the head cup bearing and the tapered junctions of orthopaedic hip implants are known to differ and the debris generated varies in size, shape and volume. Significantly less metal is lost from the taper junction between Cobalt-Chromium-Molybdenum (CoCrMo) and Titanium (Ti) components (fretting-corrosion dominant mechanism), when compared to the CoCrMo bearing surfaces (wear-corrosion dominant mechanism). Corrosion particles from the taper junction can lead to Adverse Reactions to Metal Debris (ARMD) similar to those seen with CoCrMo bearings. We used synchrotron methods to understand the modes underlying clinically significant tissue reactions to Co, Cr and Ti by analysing viable peri-prosthetic tissue. Cr was present as Cr_2_O_3_ in the corroded group in addition to CrPO_4_ found in the metal-on-metal (MoM) group. Interestingly, Ti was present as TiO_2_ in an amorphous rather than rutile or anatase physical form. The metal species were co-localized in the same micron-scale particles as result of corrosion processes and in one cell type, the phagocytes. This work gives new insights into the degradation products from metal devices as well as guidance for toxicological studies in humans.

## Introduction

In total hip arthroplasty, a stem is inserted in the medullary canal of the femur and connected to a spherical head via a Morse taper (a cone on a cone)^1^ designed to prevent motion between the trunnion and the bore and avoid fluid ingress into the metal junction. However poor surgical assembly, cyclic loads, different material combinations and the composition of the surrounding fluid may induce micro motions at the taper resulting in disruption of the passive protective oxide layer leaving the underlying metal exposed and susceptible to corrosion.

The products of metal degradation released into the periprosthetic tissue can evoke a series of inflammatory responses termed adverse reactions to metal debris (ARMD). This has widely been associated with metal-on-metal (MoM) hip resurfacing (HR) implants with Cobalt-Chromium-Molybdenum (CoCrMo) bearing surfaces (and no taper junctions^2–4^) that primarily mechanically wear at the articulating surfaces. Over the last few years, an increasing number of failures secondary to ARMD from the taper junction between CoCrMo and Titanium (Ti) components, have been reported^5–7^.

Modularity or the availability of various implant sizes at the head/neck junction is well established in modern total hip arthroplasty providing the orthopaedic surgeon a more patient-tailored solution and component-targeted revision surgery^8, 9^, more recent is the introduction of dual-taper implants^10^.

**Figure 1:**
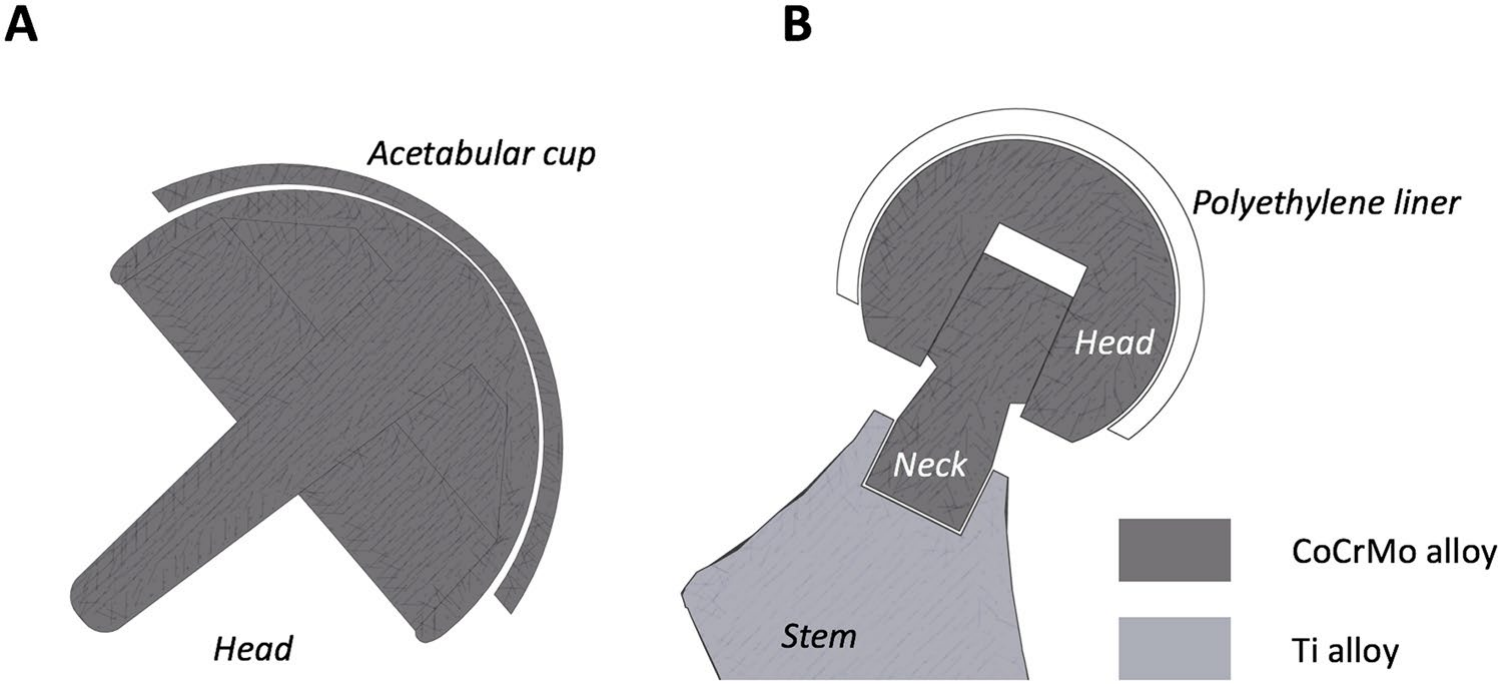
Schematic cross-sectional images of (**A**) a MoM hip resurfacing implant with both bearing surfaces made of CoCrMo alloy; no taper junction or mixed alloy coupling is present; and (**B**) a Metal-on-Polyethylene (MoP) dual-taper implant with a CoCrMo/Ti interface between neck and stem junction; no MoM bearing surfaces are present.

In dual-taper designs there is an additional interface which connects neck and stem via a Morse taper, designed to provide surgeons a bespoke solution to match the patient’s anatomy, Fig. 1.

This second junction (neck/stem) can be subjected to an increased lever arm moment when compared to the head/neck taper and therefore higher bending and torsional stresses that may explain the greater damage that is frequently reported^11–13^. For this reason, dual-taper hips with non-MoM bearings provide a unique insight into corrosion products from implants for they are known to corrode heavily at their neck-stem taper junction with negligible metal debris from the bearing^14–17^.

Synchrotron analysis of *in-vivo* intracellular wear particles is key to the understanding of clinically significant tissue reactions of different classes of implants associated with ARMD. Technological advancements have made it possible to image metals in mammalian tissue with submicron resolution without damaging the sample. This is of particular significance as it allows the amount and oxidation state of trace elements in intra-cellular compartments to be determined.

Previous work has shown that the exact chemical makeup of metals in tissue surrounding metal on metal (MoM) hip resurfacing (HR) implants is Cr (III) phosphate, Co metal and Co (II) attached to organic ligands^18^. Although it is well established that the quantity of metal released from the bearing is significantly higher than that from the taper junctions and that the debris differs in size and shape^19, 20^, little is known about the form of metal products from corroded tapered interfaces. Recently, there have been reports looking at the corrosion products from taper junctions^20, 21^, their histopathological characterisation^22, 23^ however there is no study that has used synchrotron methods to accurately evaluate their oxidation state.

In this study, we aimed to better understand the speciation of the metal released from corroded CoCrMo/Ti taper junctions by determining: (1) the exact chemical form (valency and molecular composition) of the metal particles in the tissues surrounding corroded dual-taper hips, (2) which cells contain the metal species and (3) how these results compare with those from MoM HRs.

## Materials and Methods

### Study design and patient selection

We prospectively collected and analysed periprosthetic soft tissue taken during revision surgeries of dual-taper hip replacements of a single design retrieved secondary to ARMD. Our work was approved by the institutional review boards of the Riverside Research Ethics Committee and all experiments were performed in accordance with relevant guidelines and regulations. Informed consent was obtained from all subjects or their authorized representatives prior to any study procedures. The hip prostheses consisted of Metal-on-Polyethylene (MoP) bearings with 28 mm CoCrMo alloy heads. All were paired with Rejuvenate stems (Stryker, Howmedica Osteonics Corp., NJ, USA), which consisted of a Ti-based alloy (TiMoZrFe) stem featuring an exchangeable CoCrMo neck. The Food and Drug Administration (FDA) approved this design in November 2009 and in June 2012 the manufacturer initiated a voluntary recall due to high early failure rates^24^. For this specific design, survivorships of 69.3% at two years^12^ and of 40% at four years^25^ have been reported. It has been shown that mechanically assisted corrosion is the predominant mechanism for metal release from junction between the stem and neck.

**Figure 2:**
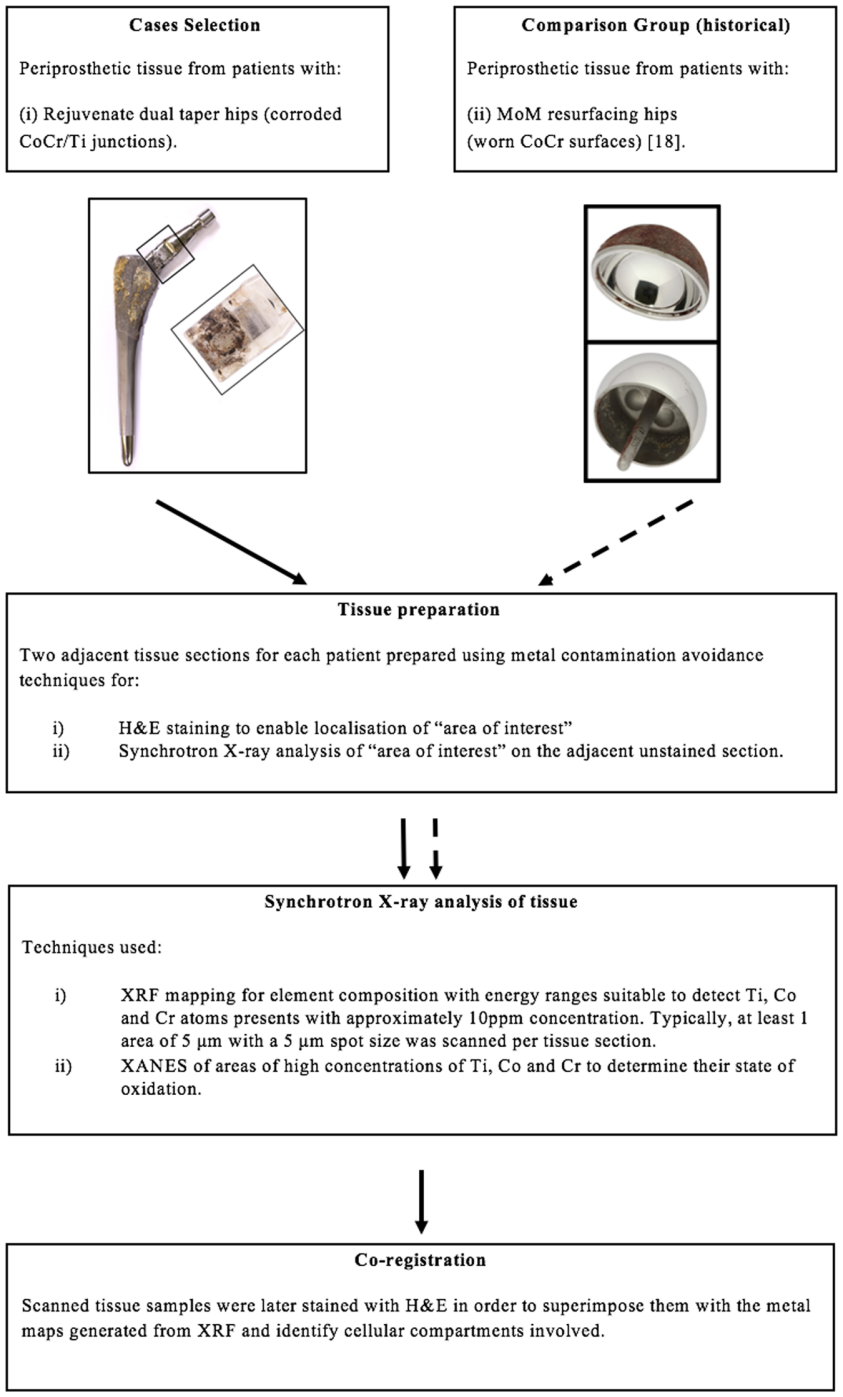
Flow chart summarising the study design.

The same methodologies used in the investigation of metal species derived from MoM HRs were applied in this study and the results compared^18^. For the first time here, the slices scanned with X-rays were then stained with H-E to performed superimposition with metal maps. This enabled us to combine both metal analysis and cell features in one image. Study details are presented in the experimental flow diagram, Fig. 2.

**Figure 3:**
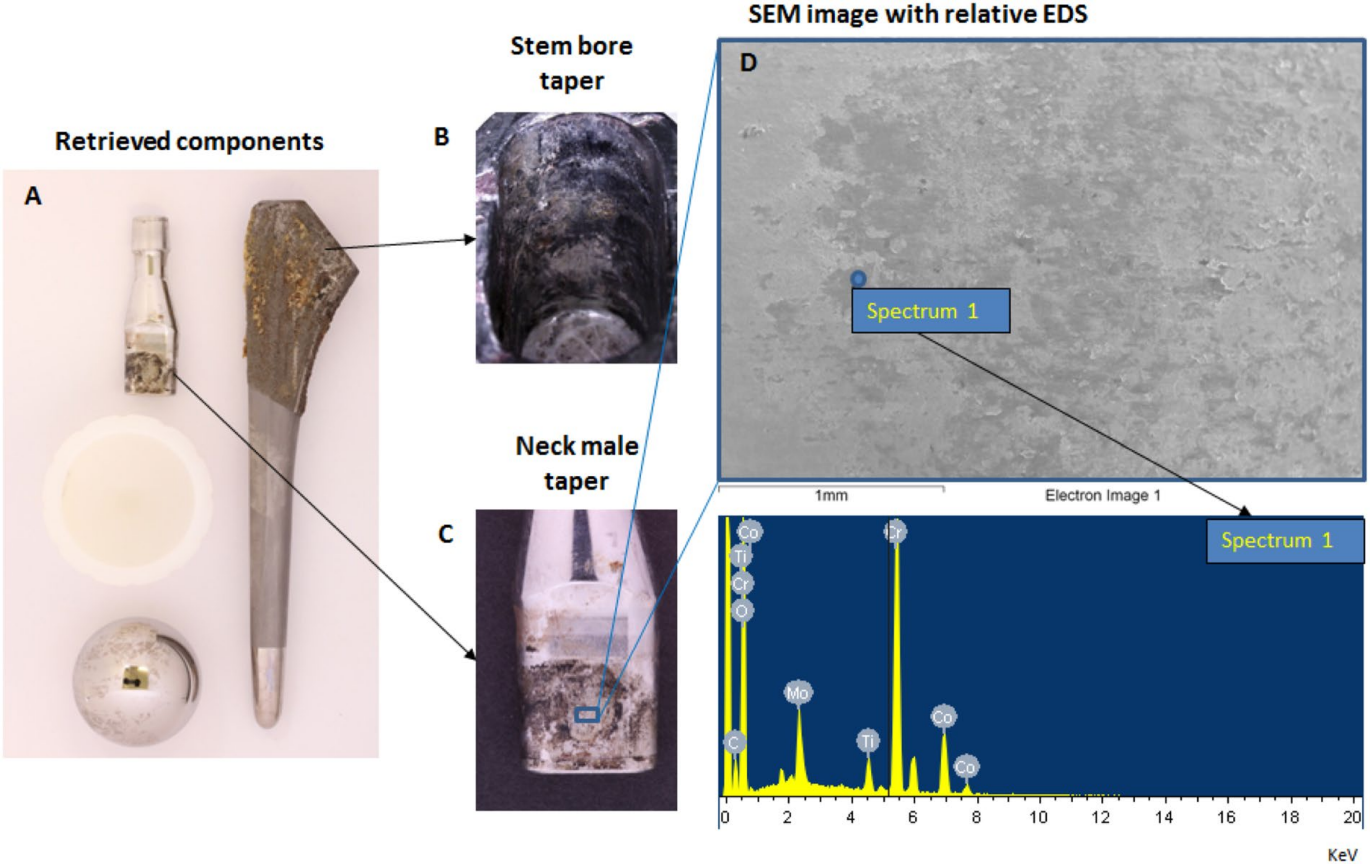
Image of a retrieved hip implant investigated in the study featuring metal-on-polyethylene bearing type (**A**). Both stem bore (**B**) and neck male (**C**) taper surfaces showed gross signs of corrosion in the engagement segment, characteristic representative of the whole cohort. Elemental analysis (**D**) confirmed corrosion as chromium rich deposits were found composing the black debris; traces of Ti were also present as a consequence of metal transfer from the stem to the neck.

On visual inspection at the time of revision, all implants demonstrated severe corrosion at the neck-stem junction, Fig. 3.

None of the implants showed signs of corrosion at the head-neck taper junction or bearing surfaces. This finding has been reported in other studies^16, 17, 26–28^.

The median (range) age of the subjects at primary implantation was 61 (47–74) years and the median period of implantation was 42 (23–53) months. Clinical data are reported in Table 1.

**Table 1:** Patient demographic and clinical data of tissue analysed.

Patient ref. number	Gender	Age at Primary	Months to failure	Co [μg/L]	Cr [μg/L]
#1*	Female	55	23	19	3
#2	Female	47	42	7	1
#3	Male	63	40	9	2
#4	Female	61	40	6	1
#5	Male	58	50	2	1.9
#6	Male	67	53	NA	NA
#7	Female	74	45	1.5	1.3

NA: not available; *Bilateral.

### Preparation of periprosthetic tissue and histological examination

Multiple tissue samples were collected from the hip capsules and bursal synovium of patients undergone revision surgery due to ARMD confirmed by magnetic resonance imaging modality. The number of surgical specimens per patient, usually between 3 and 6, varied depending on the case and surgeon. Specimens were fixed in 10% neutral buffered formalin after being subjected to extensive sampling at macroscopic examination. A number of histological sections were unsuitable for metal analysis due to presence of significant amounts of necrotic tissue and no identifiable particle content in the deep-seated band of the macrophagic/lymphocytic infiltrate.

Formalin fixed paraffin embedded tissue were sectioned at 3–4 µm slices. As in previous experiments^18, 29^, a metal contamination avoidance (MCA) procedure was performed^30^ to minimize chances of tissue contamination and/or of alteration in the distribution and chemistry of implant-related debris. For each sample, two adjacent sides of the same tissue were cut and dewaxed by immersion into xylene and alcohol solutions^29^. Histological sections were stained with haematoxylin-eosin (H-E) for cellularity and particle content. This procedure allowed a better view of the architecture of the section and aided better selection of areas of interest for the synchrotron beam mapping. The unstained section was used for the actual synchrotron analysis.

All sections were mounted on high purity silica slides. In some cases, the corrosion products were present focally or in only one fragment of the tissue in the block, a careful screening of the entire section was made.

### Synchrotron methods

The synchrotron work was conducted in the microfocus spectroscopy beamline (I18) at the Diamond Light Source, Harwell Science and Innovation Campus (Didcot, UK)^31^. The study design included two types of experiments; µX-ray Fluorescence (XRF) mapping of the tissue samples and µX-ray absorption spectroscopy (XAS) with a typical beam profile of 3 × 3 μm providing a resolution at a length scale similar to that of individual cells.

#### Synchrotron µ-XRF (metal mapping of the tissues) and co-registration

µX-ray Fluorescence mapping of the tissue samples is based on the intrinsic fluorescent properties of each element and is therefore element specific. Approximately a 500 × 500 μm region was mapped using an incident X-ray energy of 10.5 keV and a 6 element Si drift detector (SGX Sensortech) with Xspress-3 processing electronics. More than one region per each tissue examined was scanned to in order to confirm the elemental findings. The map data were analysed for various metal elements including Co, Cr and Ti in the program PyMCA^32^. The mean and median values for these elements were not normalised to beam intensity but displayed as raw counts. For each map point, Ti, Co and Cr intensities were plotted against each other to identify any patterns in the data, such as isolated high intensity spots or correlations in the element distribution.

In order to identify which cells and cellular compartment were involved with the processing of the host particles, we co-registered the sections scanned by the beam with the resultant metal maps. H-E staining is ideal for identifying cell features in tissues and localizing the areas of interest for scanning, but it can interfere with the synchrotron analysis. In fact, the stain contains a wide range of metal contaminants that would affect the metal mapping. For this reason, unstained adjacent slices were used for the synchrotron analysis. The beam does not severely damage the tissue but it leaves behind traces seen as horizontal lines visible on high magnification light microscopy. The slices scanned with X-rays were then stained with H-E to perform superimposition with metal maps. This enabled us to combine both metal analysis and cell features in one image, to obtain a visual picture of the elemental composition in the specific investigated tissue sample.

#### Synchrotron micro X-ray absorption near edge structure µXANES

µXANES was used to determine the form of the metals in the tissues. Around 50 spectra at the Cr K-edge, 20 spectra each at the Co and Ti K-edges were recorded at various points identified from the µXRF maps. At some points two or more spectra were recorded to look for evidence of beam damage. Data reduction and XANES analysis were performed using the program Athena in the Demeter suite^33^.

#### Ti, Co and Cr standards for synchrotron micro-XAS

Data for the Co and Cr spectral standards for the elements under in investigation have previously been published along with details of their preparation^18, 34^. Transmission data for the anatase and rutile, polymorphs, of TiO_2_ was provided by Professor G Sankar (UCL). All the spectra collected during different experiments were aligned using Ti, Cr and Co foil spectra recorded in transmission, setting the edge position of these foils as 4966, 5989 and 7709 eV, respectively.

### Data availability

The datasets generated during and/or analysed during the study are available from the corresponding author on reasonable request.

## Results

### Histological analysis

Histological examination of the seven periprosthetic tissues samples recovered from patients with dual-taper implants showed corrosion products present within macrophages and in the extracellular connective tissue. The corrosion particles with a unique greenish appearance distinctively differentiate from the conventional black metallic debris^22, 35^.

**Figure 4:**
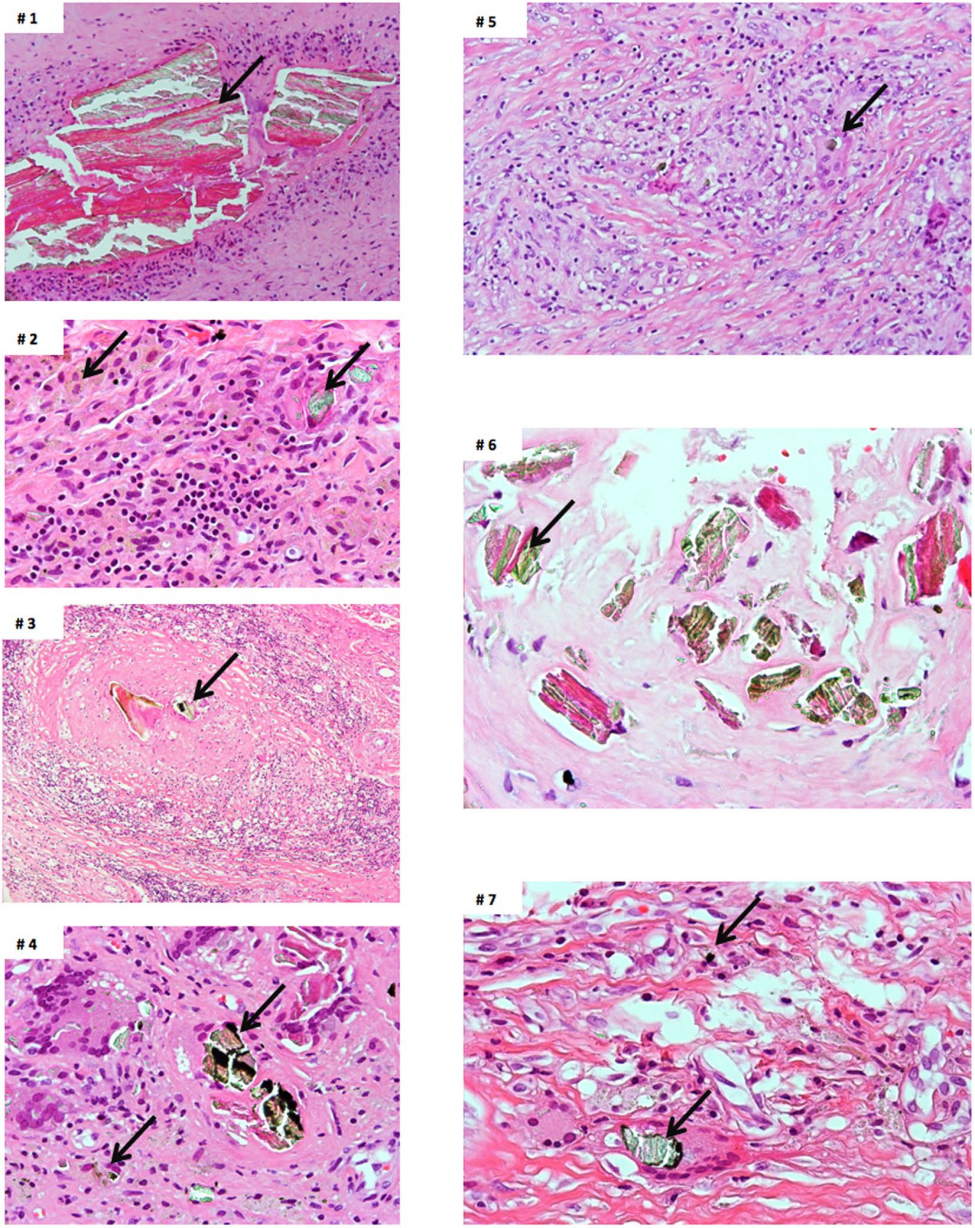
Microphotographs of the seven cases showing: (1) large corrosion aggregate deposits of greenish appearance surrounded by macrophages (arrow), (H-E × 200); (2) corrosion particulate material within macrophages (arrows), a lymphocytic inflammatory component is focally present, (H-E × 400); (3) corrosion particulate aggregate surrounded by macrophages (arrow), (H-E × 200); (4) particulate material engulfed by foreign body-type giant cells (arrows), (H-E × 400); (5) foreign body-type macrophagic reaction (arrow showing corrosion particle in giant cell) (H-E × 200); (6) large particles (arrow) within fibrous tissue (H-E × 400); (7) foreign body-type macrophagic reaction showing large and small particles (arrows) engulfed by giant cells; (H-E × 400).

The reaction to the particles was predominantly composed of macrophages with numerous scattered multinucleate foreign body-type giant cells seen around large particles of corrosion material. In addition, most cases had a predominantly perivascular lymphoplasmacytic inflammatory infiltrate. No acute inflammatory component to suggest infection was seen in the cohort. Cases are presented in Fig. 4.

### Metal mapping of the tissues (micro-XRF) and co-registration

**Figure 5:**
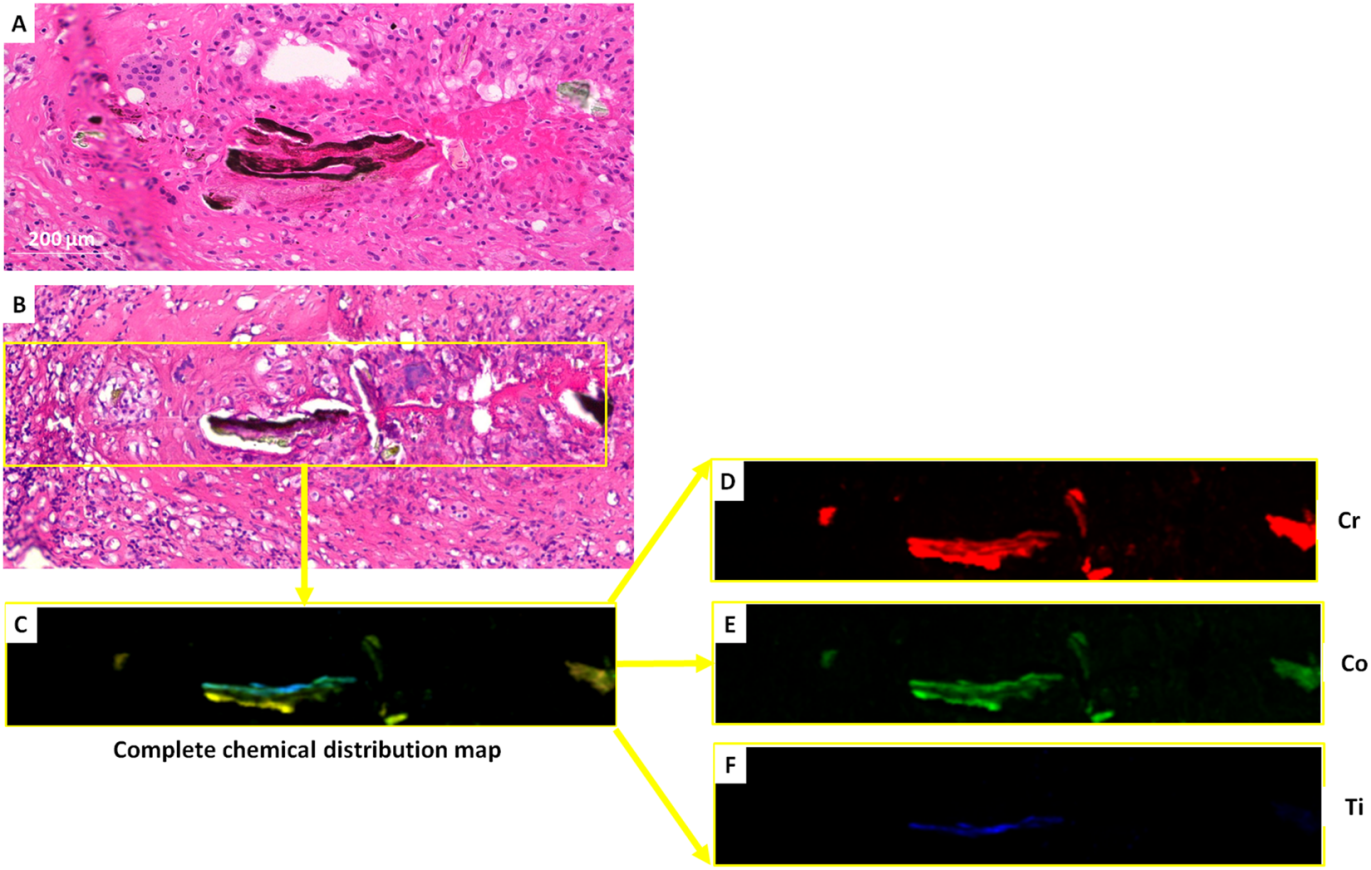
Light microscopy image of a section (**A**) from patient #7 with viable macrophages, giant cells, and cuffing of lymphocytes. Image (**B**) is the adjacent section, stained with H-E, after exposure to the Diamond Light Source. The complete chemical distribution map (**C**), showed that Cr (in red), Co (in green) and Ti (in blue), were co-localized.

**Figure 6:**
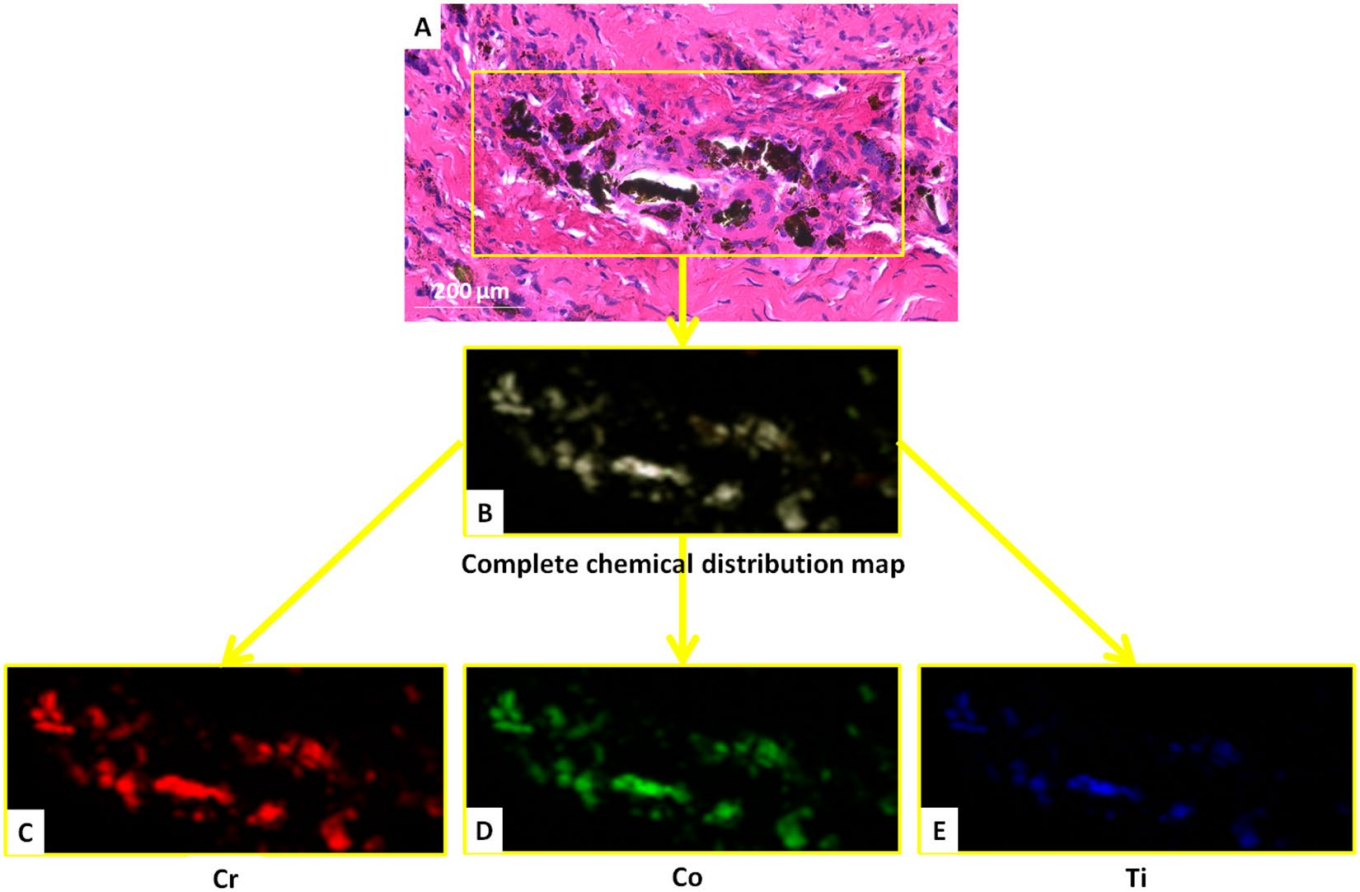
Light microscopy image of an H-E stained section from patient #2 after (**A**) exposure to the Diamond Light Source showing the cellular scanned area (yellow box) and corresponding complete chemical distribution map (**B**) revealing Cr (**C**), Co (**D**) and Ti (**E**) co-localized in the same particles.

**Figure 7:**
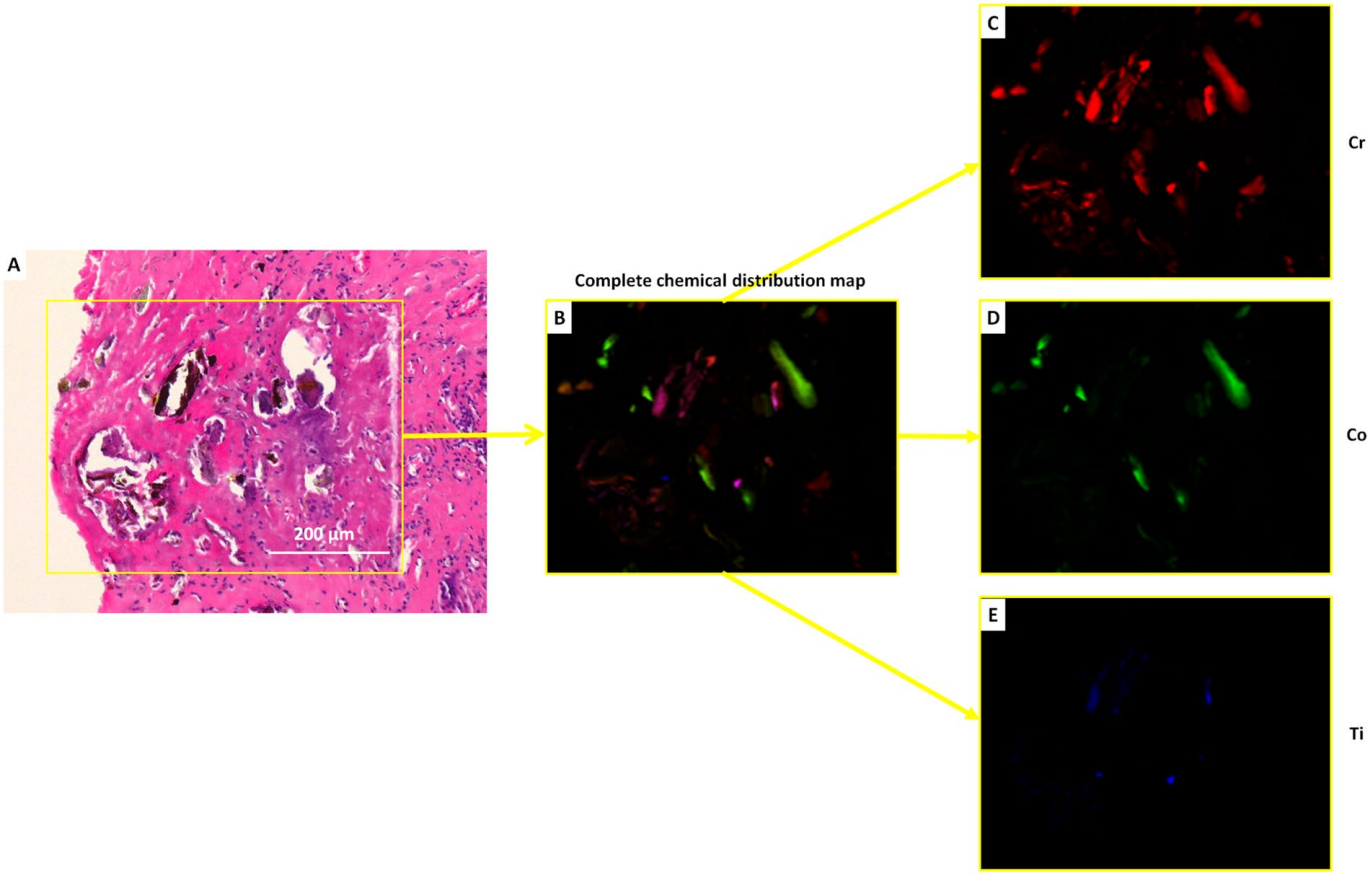
Light microscopy image (**A**) of the H-E stained sections from patient #4 after exposure to the Diamond Light Source and corresponding chemical map (**B**) containing all the elements. (**C**) Shows the Cr map, (**D**) the Co map and (**E**) the Ti map. Co-registration of metal maps with histology revealed that particulate material was lodged in the connective tissue of the capsule and/or neo-synovium with lining by giant cells whereas smaller particles were engulfed by the giant cells and further break-up into smaller fragments phagocytized by the macrophages.

The predominant metal species in the XRF maps was Cr, followed by Co and Ti. Sections exposed to the beam, H-E stained, showed that the location of metal was both extra and intra cellular. Cr, Co and Ti were co-localized in one cell type, the phagocytes: macrophages and giant cells but not seen in the fibroblasts and lymphocytes present in the tissue sections studied. Representative XRF maps with relative sections are shown for patients #7, #2 and #4 in Figs 5, 6 and 7 respectively.

### Synchrotron micro X-ray absorption spectroscopy (XAS) and X-ray absorption near edge structure (XANES)

#### Cr XAS/XANES

**Figure 8:**
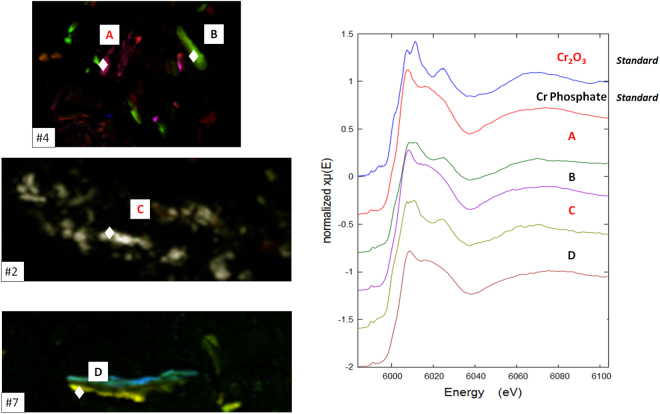
Cr XANES of representative points for patients identified as #4, #2 and #7 and from Cr_2_O_3_ and CrPO_4_ standards.

Unlike previous Cr XANES studies of tissue from around failed MoM CoCrMo hip implants^18^, we observed two types of oxidised Cr spectral types in approximately equal number. One of these types of spectra corresponds to the XANES of CrPO_4_ previously observed, while the other to the XANES of Cr_2_O_3_, Fig. 8.

There was a correlation between significant Ti concentration in the tissue and the Cr XANES spectrum being similar to Cr_2_O_3_. The Cr_2_O_3_-like spectra were from points of significant Ti concentration while there was no significant Ti where the “Cr (PO_4_)” XANES points were. No XANES indicating metallic Cr was observed.

#### Co XAS/XANES

**Figure 9:**
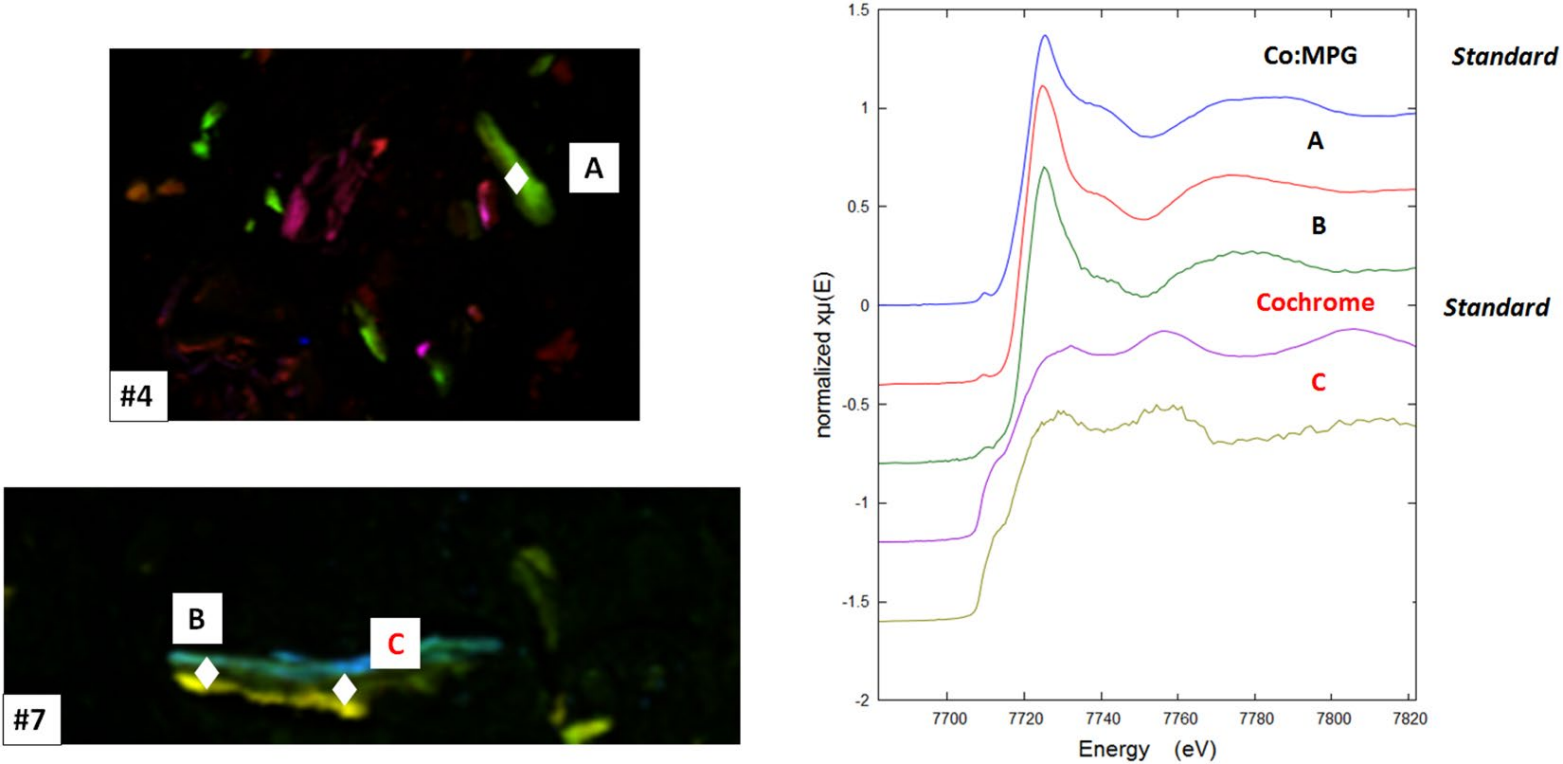
Representative Co XANES spectra for patients #4 and #7 and for CoCr and a Co:MPG complex standards. Hence most of the Cobalt was found to be in oxidation state (II) in a complex organic coordination environment (Co:MPG).

Almost all the Co XANES spectra obtained demonstrated a similar pattern, Fig. 9. These spectra were similar to XANES of Co (II) with organic ligands in an octahedral environment e.g. the cobalt N-(2-mercaptopropionyl) glycine complex (a Co-peptide mimetic, Co:MPG). There were two exceptions from sample #7 with Co spectra being similar to spectra from Co in Cochrome, hence in a metallic state as seen in MoM HRs^18^.

#### Ti XAS/XANES

**Figure 10:**
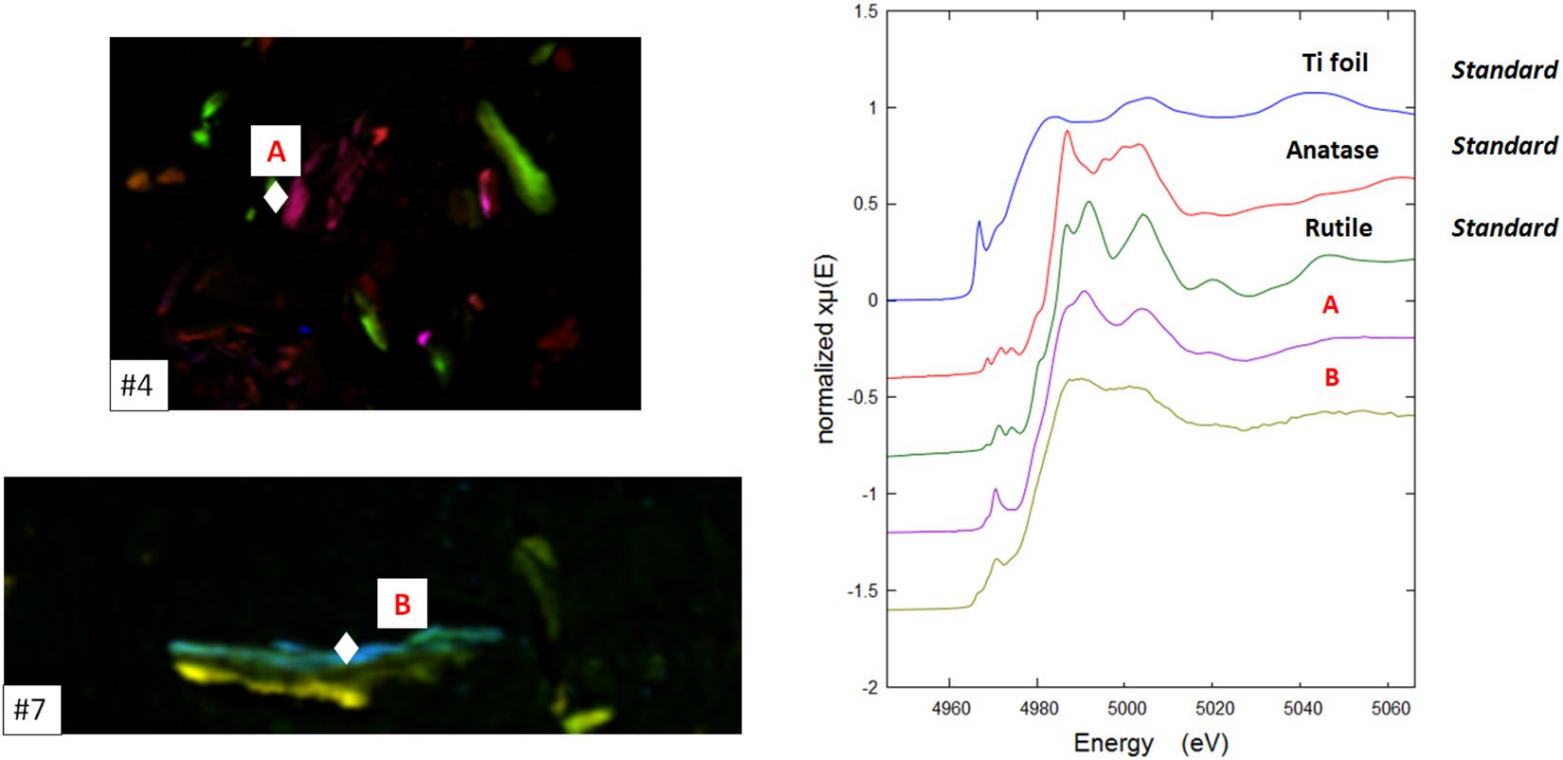
Ti K-edge XANES of standards Ti foil, Ti rutile and anatase and representative XANES for patients #4 and #7.

The Titanium XANES spectra were all representative of oxidised titanium species. The majority of the Ti spectra (n = 15) from different patient tissues were similar (Fig. 10A) however a few from #6 and #7 were somewhat different but similar to each other, Fig. 10B.

Unlike in previous reports on Ti in tissue studies^36^, neither type of spectra was similar to the XANES of either of the common polymorphs of TiO_2_, anatase or rutile. The small pre-edge peaks, which represent transitions to d orbitals, in the spectra of anatase and rutile are very different to the pre-edge of Ti in the tissue. This higher pre-edge peak in spectra of type #Ti1 indicates much of the titanium may be a disordered oxide species^37^; it bears a resemblance to the spectra of amorphous TiO_2_^38^.

## Discussion

**Figure 11:**
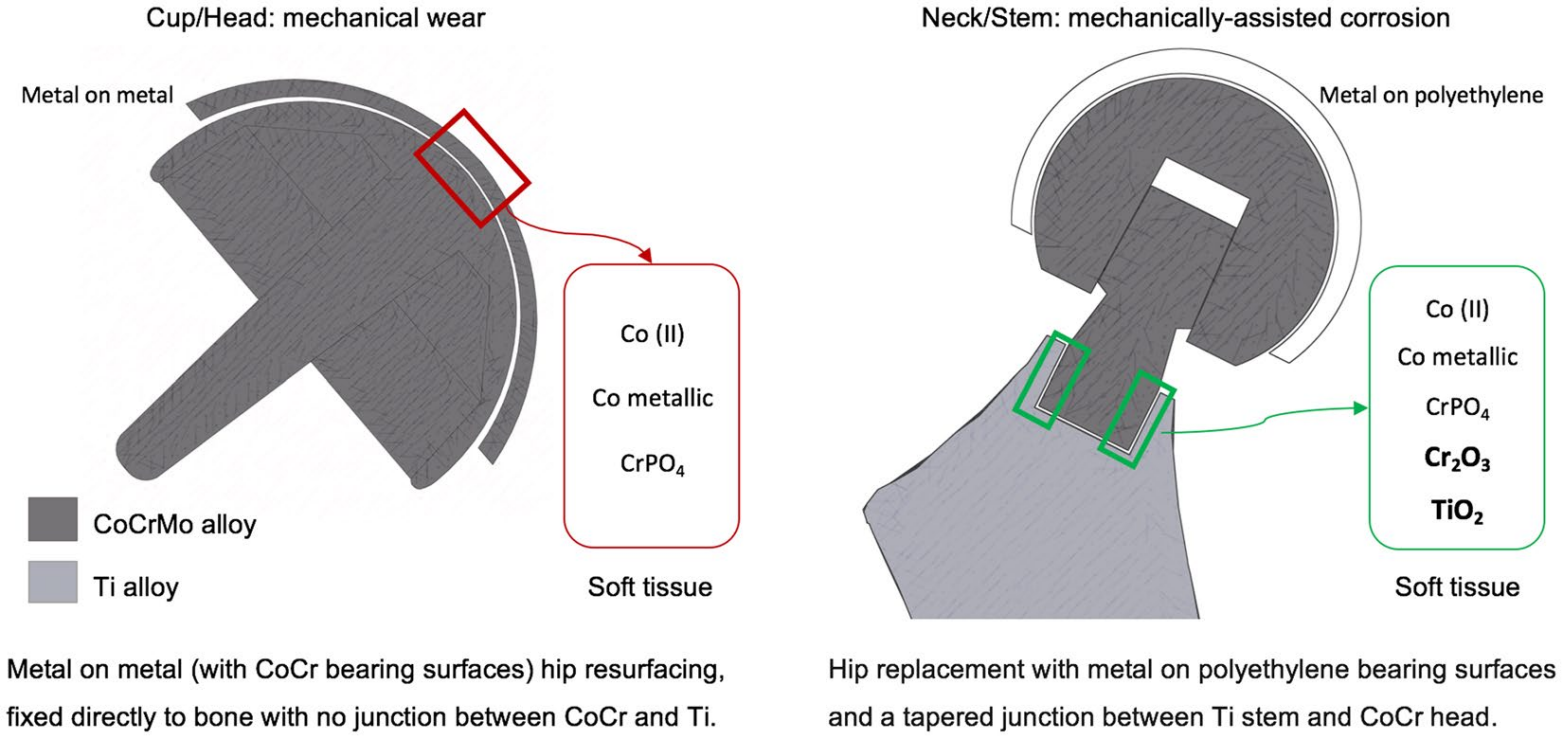
Summary of the differences in the metal speciation into tissues surrounding dual-taper hips (current study) and MoM hip resurfacings (comparison group [18]). The dual-taper hip has a metal-on-polyethylene bearing type, head and neck made from ASTM F75 CoCrMo alloy and stem made from ASTM F1813: 01 wrought Ti-12 Molybdenum-6 Zirconium-2 Iron alloy. The CoCrMo neck/TMZF stem junction resulted to be heavily corroded at the time of revision. The MoM hip resurfacing implant has both bearing surfaces made from ASTM F75 CoCrMo alloy with a ratio of Co to Cr of 2:1, no taper junction or alloy coupling is present.

This is the first study to use synchrotron radiation to chemically characterise the implant-derived metal species in human tissue surrounding corroded taper junctions. This is important for toxicology studies that will hopefully result in a better understanding of why some patients have devastating adverse responses to metal debris. Comparison between these results and those found for MoM HRs synchrotron analysis are summarised in Fig. 11.

The novel findings from this study are: (1) the speciation of chromium into chromium oxide (Cr_2_O_3_-like species) which has not been seen previously in MoM studies; (2) the correlation between significant titanium concentrations in the tissue and Cr_2_O_3_, with no such correlation between titanium and CrPO_4_, possibly explained by the electrochemical interaction between CoCrMo and Ti alloys secondary to mechanically assisted fretting and crevice corrosion; (3) the speciation of titanium, into an amorphous Ti dioxide, showing a dissimilar spectrum to the common anatase or rutile reported in previous human tissue studies^36^. Due to its perceived biocompatibility, it is estimated that more than 1000 tonnes of Ti is implanted in patients every year in the form of medical devices^36^. However, it has been showed that when corrosion takes place on the surfaces of such alloy, the products of degradation can cause adverse tissue response^39^ via production of pro-inflammatory cytokines^40^. Effects of Ti dioxide on human tissue are not clearly understood. Our data supports the evidence that it could have an active role in eliciting a pathologic response in periprosthetic tissue. Moreover, it is noteworthy (4) the particle composition complexity; in other words, the co-localisation of the elements, with chromium showing greater affinity with titanium than cobalt. These differences suggest a causal relationship between failure rates and modes of failure.

The substantial variations between the present investigated implants and the previous MoM hip resurfacings^18^ reside in the material combination and the type of junction and this is crucial to relate cause (implant design) and effects (tissue reaction).

The dual-taper hips had MoP bearings and CoCrMo/Ti taper junctions (neck-stem). Mixed metal alloy combinations have been shown to increase fretting corrosion. The potential difference between two metals in a conductive solution results in an electron flow between the metals usually resulting in accelerated corrosion of the more susceptible alloy, in this case the CoCr alloy over the Ti alloy^41^. Whereas the resurfacing hips predominantly release metal through wear-corrosion at the bearing surfaces sliding across each other and they are made of the same alloy.

It is documented that MoM bearings wear substantially more than Morse taper junctions and the material released differs in terms of size, shape and therefore cellular responses to them^22^. Tribocorrosion at the bearing surfaces produces debris in the range of nanometres^42, 43^ that are predominantly round in shape^20^. We found micro-sized aggregates of corrosion particles with plate-like structure in tissue surrounding dual-taper hips with agglomeration of the metal species as result of mechanically assisted fretting corrosion not seen in tissue surrounding failed MoM HR implants and in agreement with previous reports^20^.

Comparison of large studies reveals that patients with non-MoM dual taper hips with CoCrMo alloy necks and Ti alloy stems have lower whole blood metal ion levels compared to patients with resurfacing or large diameter head MoM implants^6, 44^. However, the release of species from taper junctions, is associated with elevation of cobalt in excess of chromium in the blood stream rather than the bearing surfaces^45^. This is thought to be the result of the precipitation from solution of Cr which, due to the taper design, tends to be retained in the black corrosive surface debris whilst the more soluble Co is readily excreted^46^. Cobalt solubility is also confirmed by the rate at which Co and Cr levels decrease in patients after removal of the implants^47^. Metal ions tend to bind to proteins and are transported into the body via the circulatory system, whereas particles build into the periprosthetic tissue, inducing a local tissue response. In this study the most abundant metal species found in the soft tissues surrounding the dual-taper implants was Cr, despite the composition of CoCrMo alloy, supporting that corrosion at the junctions is the dominant mechanism of metal release in these hips; in agreement with retrieval studies comparing metal ion levels between MoM resurfacing and taperd THAs^46^.

Our results indicate that the metal species and their complex configurations may have a significant role in eliciting a pathologic response in tissue, hence the early failure of metal devices. The findings provide guidance for toxicological studies on wear/corrosion particles both *in-vitro* and *in-vivo*, mechanisms that stimulate the host response as well as the cellular processes involved in the pathogenesis of ARMD and set the basis for future work on the interaction between the species found and proteins, known to initiate the inflammatory cascade. They also provide useful information regarding future pre-marketing tests of orthopaedic implants, generally focussed on wear debris generated during tribocorrosion in hip simulators which, in light of these findings, may be an oversimplification of the actual configuration of the particles generated into the body.

Recent studies have reported on the finding of Cr oxide in metal particles surrounding tapered implants^20, 21^, however this is the first to use synchrotron radiation to confirm it. Synchrotron µXRF is more accurate in interrogating metal in human tissues compared to electron micro-probing. The typical detection limit of electron probe microanalysis is 100 parts per million (ppm) and this can be improved to 10 ppm in optimal specimen samples^48^. For transition metal in tissue samples XRF has a detection limit of hundreds of parts per billion^49^. The methods used enabled mapping of large areas of many tissue samples followed by repeated chemical analysis of element, valency and nearest atomic neighbours that is widely accepted as precise and accurate. Additionally, synchrotron X-ray analysis causes minimal “beam damage” and allows subsequent staining to establish the cell types and tissue architecture in relation to the location of the metal species found.

Around 50 spectra at the Cr K-edge, 20 spectra each at the Co and Ti K-edges were recorded at various points identified from the µXRF maps. Our findings are robust as they are consistent within the sample set and fitted well to the spectroscopic curves generated by the standards.

We acknowledge limitations with the current study. Firstly, elements such as Mo and Z were excluded from the analysis as they require a different X-ray energy than that set for Co, Cr and Ti to be studied, iron (Fe) instead was selectively excluded because it would have been impossible to distinguish between Fe deriving from the implant and that naturally present in tissue. Chemical interactions between Mo, Z and the remaining elements cannot be excluded and could play a role in the formation of the metal species found^50^. Secondly, it was not possible to discern if the elements clustered in the same particles were bound together forming a “metal entity” *in-vivo*. Thirdly, patient specificity within the sample set such as local tissue pH as well as pre and post-operative physical activity known to affect the *in-vivo* performance of metal implants, could not be taken into account.

“Metal poisoning” was thought to be a problem limited to the 1.5 million people implanted with metal-on-metal hips, which release CrPO_4_, as shown in our synchrotron study of human tissue^29^. Although successfully used for many decades, over the last few years hip implants that have a Ti-CoCrMo junction have been advocated to be responsible for the development of adverse tissue reactions from metal debris, probably a result of modern designs and materials that have increased the release of metals / changed their species, to a level / form that causes human tissue reactions.

The stems investigated in the current study were composed of a beta titanium alloy known as TMZF (Ti-12Mo-6Zr-2Fe). It is proprietary alloy from a single manufacturer (Stryker) implanted in over 30,000 patients. This is of a different composition to the alpha-beta titanium alloys Ti-6Al-4V (which are used in the majority of orthopaedic implants), albeit both featuring more than 80% Ti content. Further work is required to understand the similarities or differences in the interactions between the two alloys and CoCr. However it is of note that the retrieval evidence of severe corrosion seen in the current study, using TMZF, is similar to that previously reported when Ti-6Al-4V alloys have been used.

## Conclusions

3 million people have hip implants every year. Understanding corrosion at taper junctions and the pathogenesis of the biological response leading to implant failure is of significant clinical importance. This is the first study to co-register histology and metal distribution maps, to explore the potential synergistic effect of CoCrMo with Ti alloy and the speciation of Ti; a crucial step in the process of understanding the toxicological effects of metal degradation.

We demonstrated for the first time the presence of Cr in the Cr_2_O_3_ species in soft tissue surrounding corroded taper junctions in addition to CrPO_4_ found when metal bearing surfaces sliding across each other. Interestingly, unlike in previous reports, Ti was present as TiO_2_ in an amorphous rather than rutile or anatase physical form.

This work is of relevance to the understanding of the science of metal-metal interactions in the human body and its clinical significance. The findings are not only applicable to the field of orthopaedics, but potentially more broadly to the medical fields including metal devices.
